# Exploring the contributions of two glutamate decarboxylase isozymes in *Lactobacillus brevis* to acid resistance and γ-aminobutyric acid production

**DOI:** 10.1186/s12934-018-1029-1

**Published:** 2018-11-19

**Authors:** Changjiang Lyu, Weirui Zhao, Chunlong Peng, Sheng Hu, Hui Fang, Yujiao Hua, Shanjing Yao, Jun Huang, Lehe Mei

**Affiliations:** 1 School of Biological and Chemical Engineering, Zhejiang University of Science and Technology, Hangzhou, 310023 China; 2 College of Chemical and Biological Engineering, Zhejiang University, Hangzhou, 310027 China; 3 School of Biotechnology and Chemical Engineering, Ningbo Institute of Technology, Zhejiang University, Ningbo, 315100 China

**Keywords:** *Lactobacillus brevis*, GAD system, GABA, Acid resistance

## Abstract

**Background:**

The glutamate decarboxylase (GAD) system of *Lactobacillus brevis* involves two isoforms of GAD, GadA and GadB, which catalyze the conversion of *L*-glutamate to γ-aminobutyric acid (GABA) in a proton-consuming reaction contributing to intracellular pH homeostasis. However, direct experimental evidence for detailed contributions of *gad* genes to acid tolerance and GABA production is lacking.

**Results:**

Molecular analysis revealed that *gadB* is cotranscribed in tandem with upstream *gadC*, and that expression of *gadCB* is greatly upregulated in response to low ambient pH when cells enter the late exponential growth phase. In contrast, *gadA* is located away from the other *gad* genes, and its expression was consistently lower and not induced by mild acid treatment. Analysis of deletion mutations in the *gad* genes of *L. brevis* demonstrated a decrease in the level of GAD activity and a concomitant decrease in acid resistance in the order of wild-type> Δ*gadA*> Δ*gadB*> Δ*gadC*> Δ*gadAB*, indicating that the GAD activity mainly endowed by GadB rather than GadA is an indispensable step in the GadCB mediated acid resistance of this organism. Moreover, engineered strains with higher GAD activities were constructed by overexpressing key GAD system genes. With the proposed two-stage pH and temperature control fed-batch fermentation strategy, GABA production by the engineered strain *L. brevis* 9530: pNZ8148-*gadBC* continuously increased reaching a high level of 104.38 ± 3.47 g/L at 72 h.

**Conclusions:**

This is the first report of the detailed contribution of *gad* genes to acid tolerance and GABA production in *L. brevis*. Enhanced production of GABA by engineered *L. brevis* was achieved, and the resulting GABA level was one of the highest among lactic acid bacterial species grown in batch or fed-batch culture.

**Electronic supplementary material:**

The online version of this article (10.1186/s12934-018-1029-1) contains supplementary material, which is available to authorized users.

## Background

Lactic acid bacteria (LAB) comprise a heterogeneous group of commercially important microorganisms extensively utilized in a large variety of food fermentations including dairy, meat, fish and vegetable products [1]. The growth of LAB is characterized by the generation of acidic end products of fermentation, which accumulate in the extracellular environment, lowering the pH and preventing the growth of spoilage bacteria [2, 3]. This distinctive feature is the basis of widely practiced methods of food preservation via fermentation [4]. However, acidification also has a detrimental effect on the cellular physiology of LAB, reducing the activity of acid-sensitive enzymes and damaging proteins and DNA. As the roles or functions of LAB are directly related to their acidogenicity (ability to produce acid at low pH) and acidurance (capacity to function at low pH) [5], it is important to understand how LAB sense and subsequently adapt to the acidic environment and what contributes to these adaptations.

Living cells are critically dependent on cytoplasmic pH homeostasis because most proteins have distinct pH ranges within which they can function [6, 7]. To counteract the acidification of the extracellular environment, LAB employ a complex but efficient combination of active and passive acid resistance systems [7, 8]. Remarkably, among various types of acid responses and tolerance mechanisms, the GAD system is regarded as one of the most potent acid mitigating pathways. In this system, intracellular protons are consumed through decarboxylation of glutamate in the cytoplasm and exchange of the reaction product GABA with extracellular glutamate, which contributes to protecting cells from the acid stress encountered during food fermentation and in the gastrointestinal tract [9, 10].

The existence and mode of action of the GAD system was first examined in LAB more than 65 years ago [11]. In addition to being the major acid survival pathway was confirmed in *Lactococcus lactis* [9] and *Lactobacillus reuteri* [12], the glutamate-dependent system has also been studied in LAB due to the link between glutamate decarboxylation and ATP synthesis through the generation of a proton motive force (PMF) [13]. Moreover, by combining the attributes of a GRAS status of food fermenting LAB and the physiological functions of GABA in the mammalian central nervous system, such as hypotensive, hepatic encephalopathy and diabetes reduction [14–16], many researchers have focused on investigations into GABA-producing LAB from various viewpoints. Some of these perspectives include the genetic and biochemical characterization of GAD, and GABA production based on the medium composition or fermentation condition [17, 18].

Among a variety of reported LAB species, *Lactobacillus brevis* strains are the most frequently isolated from traditional fermented products with the highest GABA productivity [17, 19, 20]. GABA production using *L. brevis* as bacterial cell factories is therefore a focus of research [21–24]. Previous investigations have shown that in contrast to other LAB species, the *L. brevis* chromosome generally contains distinct genes encoding two biochemically identical isoforms of GAD: GadA and GadB [23, 25, 26]. Additionally, the *gadC* gene, located upstream of *gadB*, has been proposed to encode the antiporter implicated in GABA export [23, 26]. Notably, although transcriptional levels in several *L. brevis* strains are typically analyzed by measurements of relevant mRNA abundance [23, 26, 27], the physiological role and regulatory mechanism of the GAD system in this species remains largely unclear. In addition, direct experimental evidence for the detailed contribution, if any, of *gad* genes to the acid tolerance and GABA production of *L. brevis* is lacking.

Because understanding the role and regulation of the GAD system is a prerequisite for exploiting such characteristics of acid resistance and GABA production, it is necessary to gain a deeper insight of role of the glutamate decarboxylase-dependent system in the physiology of *L. brevis*. To address the above issues, in the present work we focused our attention on the high GABA-producing strain *L. brevis* CGMCC1306 [21, 28, 29], with the aim of identifying genes that may explain its ability to grow under acid conditions and its GABA production. To further enhance GABA production from *L*-monosodium glutamate (MSG), recombinant *L. brevis* strains overexpressing GAD system key genes were constructed based on a nisin-controlled gene expression system (NICE) [30]. Moreover, a two-stage pH and temperature control with substrate feeding strategy was also developed to enhance the production of GABA.

## Results

### Gene locus of GAD system in *L. brevis* CGMCC1306

**Fig. 1:**
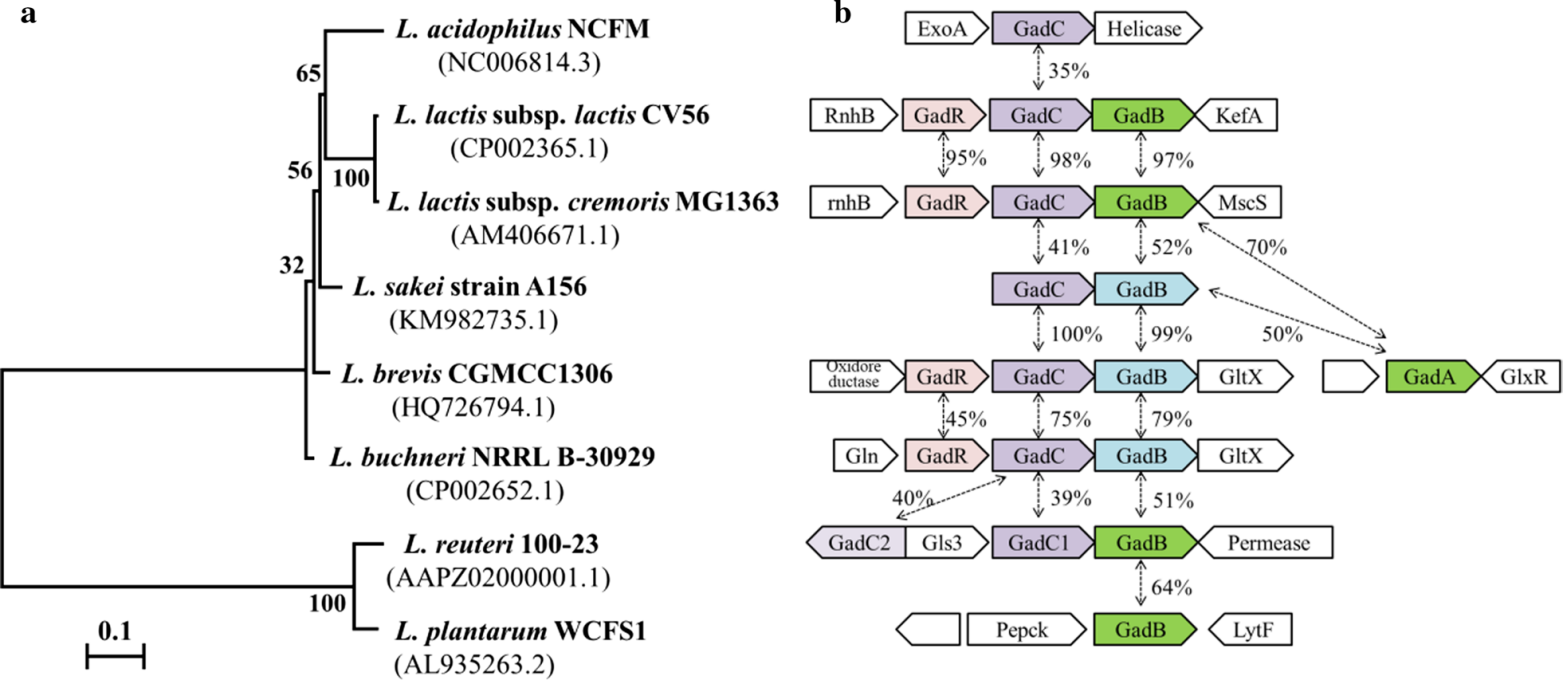
Genetic organization of the glutamate decarboxylase gene clusters in eight selected genomes of LAB strains (not drawn to scale). **a** Neighbor-joining phylogenetic tree for these LAB species based on *16S rRNA* gene sequence analysis. Bootstrap values are calculated from 1000 replications, and these values are shown at branch points (calculated by neighbor-joining, maximum likelihood and maximum parsimony methods). The bar represents 0.1 changes per nucleotide position. GenBank accession numbers are shown under the names of the strains. **b** Representation of gene loci encoding the proteins GadR, GadC and GadB in the eight strains. Numbers indicate protein identity

**Fig. 2:**
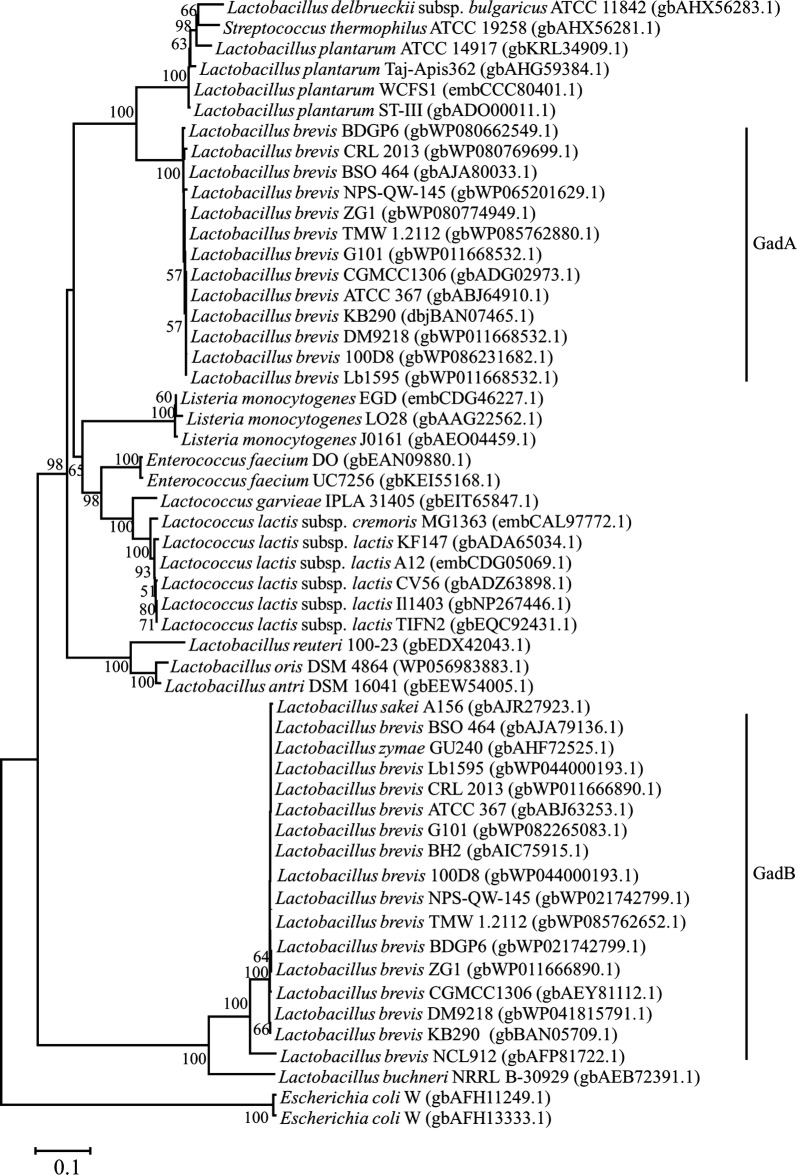
Phylogenetic tree based on deduced amino acid sequences of glutamic acid decarboxylases. Glutamate decarboxylase homologs from various representative organisms were aligned using ClustalX, and the phylogenetic tree was constructed with the neighbor-joining (NJ) method using MEGA 4.0. GenBank accession numbers are shown for all proteins. The numbers at the branches indicate supporting bootstrap values (1000 replications) for the NJ analyses. Bootstrap values above 50% are shown

The genetic determinants of the GAD system in several representative strains of LAB were characterized, as shown in Fig. 1. The gene organization present in *L. brevis* differs from the previously characterized GAD gene clusters in *L. lacti*s [9], *Lactobacillus acidophilus* [31] and *L. reuteri* [32]. In particular, sequence analysis revealed that existence of two different GAD-encoding genes (*gadA* and *gadB*) in strain CGMCC1306. Indeed, this is a common characteristic shared by *L. brevis* strains (Fig. 2), except for *L. brevis* NCL912, with only one cloned gene encoding GAD [27]. The *gadB* is linked to the glutamate/GABA antiporter gene (*gadC*), and *gadA* is located separately from the other *gad* genes. In *E. coli* [33], *Shigella flexneri* [34] and *Brucella microti* [35], *gadC* genes are preceded by *gadB*, whereas the gene order in *L. brevis* is *gadC* followed by *gadB*. The *gadC* gene is preceded by an 8-bp inverted repeat (IR; ΔG[25 °C] = − 9.5 kcal/mol) and a possible ribosome-binding site (Additional file 1). In addition, no putative promoter or terminator signals were identified in the 52-bp intergenic region between *gadC* and *gadB*, which suggests that they may form an operon, i.e., *gadCB*.

An additional gene, *gadR*, located upstream of *gadC* codes for a putative transcriptional regulator of *gadCB*. The deduced amino acid sequence is homologous (66% identify) to GadR, a possible positive regulator involved in expression of *gadCB* in *L. brevis* NCL912 [27]. Similarly, *gadR* is preceded by an 18-bp inverted repeat (IR; ΔG[25 °C] = − 18.2 kcal/mol) and a possible ribosome-binding site (Additional file 1), which suggests that transcription of *gadR* might be independent of the *gadCB* operon. In particular, the *gadRCB* gene cluster in *L. brevis* CGMCC1306 shows the same gene order and organization as in *L. lactis* subsp. *lactis* CV56, *L. lactis* subsp. *cremoris* MG1363 and *L. buchneri* NRRL B-30929. However, there is no evidence that the *gadR* gene exists in other LAB species, except for strain *L. sakei* A156, the genome sequence of which has not yet been published [36]. In line with these differences, *gadC* and *gadB* of *L. brevis* and those genes of *L. sakei* share high sequence identity but are more distantly related to these genes in *L. lactis*, *L. reuteri* and *L. plantarum*.

### Expression of the *gadCB* operon is pH-dependent

**Fig. 3:**
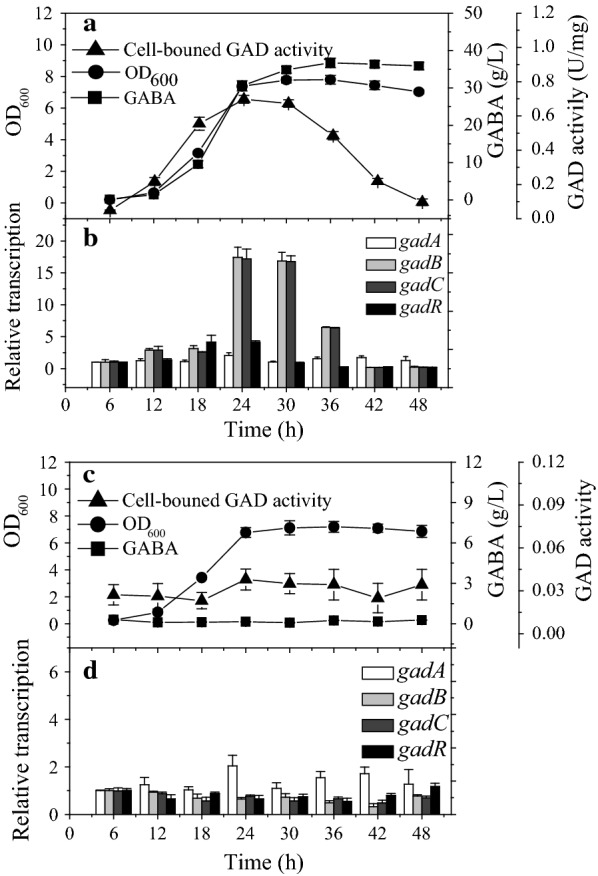
Growth and GABA production of *L. brevis* CGMCC1306 during fermentation at different constant pH values: pH 5.2 (**a**) or pH 6.8 (**c**). The relative transcription level of *gad* genes during fermentation with the pH maintained at different constant pH values: 5.2 (**b**) or 6.8 (**d**). The values presented are the means of three replicate cultures; error bars representing standard deviations may be obscured by symbols

To determine whether *gad* system genes in *L. brevis* CGMCC1306 are subject to acid-induced transcriptional regulation, a reverse-transcription quantitative PCR assay was performed in which the expression levels of all four *gad* genes were determined. When cells were grown in yeast extract peptone (GYP) medium under acidic conditions (pH 5.2), the relative expression levels of *gadR*, *gadC* and *gadB* differed by 0.2- to 17.4-fold from the control group (cells cultured in GYP medium for 6 h). The highest expression levels were found in the case of the last two genes of the *gad* cluster, *gadC* and *gadB*. Moreover, transcription of *gadC* and *gadB* was synchronous (Fig. 3b). The expression level was highest at the late exponential phase of growth. Upon further fermentation, a sharp decline in overall relative expression levels of the *gad* genes was observed from the start of the stationary growth phase. At pH 6.8, a less pronounced decrease in the relative expression levels of *gad* cluster genes was found compared to those at pH 5.2. As shown in Fig. 3d, the increased expression of *gadRCB* genes was nearly undetectable. Notably, overall expression of *gadA* gene was consistently low and did not change in response to acid (pH 5.2) or neutral (pH 6.8) conditions. These results clearly demonstrate that expression of the *gadCB* operon is pH dependent, while expression of the *gadA* gene is constitutive. In addition, the transcriptional level of *gadC* was identical to that of *gadB*, further suggesting the existence of a *gadCB* operon in this strain. Indeed, this is consistent with PCR analysis verification (Additional file 2).

To explore the relationship between *gad* gene expression and GABA levels, GABA concentrations were determined under different fermentation conditions. The results showed that pH had a significant effect on the kinetics of MSG conversion and cell-bound GAD activity. As shown in Fig. 3a, when 75 g/L of MSG was used as the substrate, 34.9 g/L of GABA was produced after 30 h of batch reaction under acidic conditions (pH 5.2). Conversely, no obvious conversion of MSG to GABA occurred in pH-controlled fermenters at a pH value of 6.8 (Fig. 3c). Overall, there was a good correlation between expression of *gadRCB* and GABA production in *L. brevis* CGMCC1306.

### GAD activity assays using deletion mutants

A positive correlation between cells possessing GAD activity and the gene expression levels of the *gadCB* operon was observed, suggesting that *L. brevis* CGMCC1306 may employ *gadCB* gene products rather than GadA for its main GAD activity. To investigate this possibility, deletions in *gadC*, *gadA* and/or *gadB* were generated using the double crossover homologous recombination method, as described previously [37]. Because different GAD activities were observed during the various growth phases (Fig. 3a), the GAD activity assay was conducted on parent and mutant strains under two conditions: cells in exponential (EXP.; 12 h) and early stationary (EARLY-STAT.; 30 h) phases (Table 1). For exponential phase cells, deletion of *gadA* resulted in a small reduction in cell-bound GAD activity; however, deletion of *gadB* in *L. brevis* led to a more significant decrease, reducing activity by approximately 16.2-fold. As expected, GAD activity was essentially eliminated in the Δ*gadA*Δ*gadB* double mutant. It is noteworthy that very low levels of activity remained in the Δ*gadC* mutant, and this unexpected result might have been caused by some degree of cell lysis resulting in extracellular GadB and GadA activity. In further experiments, a roughly similar trend was observed in early stationary-phase cells (Table 1). No activity was observed for the Δ*gadA*Δ*gadB* double mutant, and only slight activity was found for the Δ*gadC* mutant.

**Table 1:** Cell-bound GAD activity

	WT (U/g DCW)	Δ*gadA* (U/g DCW)	Δ*gadB* (U/g DCW)	Δ*gadAB* (U/g DCW)	Δ*gadC* (U/g DCW)
EXP.	218.13 ± 23.18	173.56 ± 8.75	13.44 ± 2.67	0.05 ± 0.02	0.75 ± 0.09
EARLY-STAT.	672.72 ± 22.04	605.47 ± 30.12	22.49 ± 7.43	0.08 ± 0.07	1.96 ± 0.14

Cells were collected at the exponential phase (EXP.; 12 h) and early stationary phase (EARLY-STAT.; 30 h). One unit (U) of GAD activity was defined as the mass of cells that produced 1 µmol of GABA in 1 min. Specific activity was defined as U/g dry cell weight (DCW) of cells

Analysis of a set of four *gad* deletion mutants demonstrated a dramatic decrease in the level of cell-bound GAD activity in the order of wild-type> Δ*gadA*> Δ*gadB*> Δ*gadC*> Δ*gadA*Δ*gadB* (Table 1), indicating that GadB rather than GadA was the main contributor to the overall GAD activity under this condition. Moreover, these data indicate that this strain has no third GAD that is sufficiently active under the tested conditions to substitute for GadB. These combined results strongly suggest that pH-dependent GadC and GadB play a more important role in overall GAD activity relative to GadA, at least under the conditions tested in this study. Remarkably, in contrast with the cell-bound GAD activities measured in these mutants, the cytoplasmic GAD activity of *L. brevis* Δ*gadC* was similar to that of the wild-type strain (Table 2). This result demonstrates that GAD functions in strict association with a cognate GadC, which is localized to the cell membrane and provides a selective gate for the entry of glutamate and exit of the decarboxylation product GABA [38].

**Table 2:** Cytoplasmic GAD activities of *L. brevis* strains

	WT (U/mg of protein)	Δ*gadA* (U/mg of protein)	Δ*gadB* (U/mg of protein)	Δ*gadA*Δ*gadB* (U/mg of protein)	Δ*gadC* (U/mg of protein)	Δ*gadB*: pMG36e-*gadB* (U/mg of protein)
EXP.	1.31 ± 0.09	1.24 ± 0.16	0.19 ± 0.08	0.04 ± 0.02	1.35 ± 0.17	3.82 ± 0.27

Cells were collected at exponential phase (EXP.; 24 h) and then lysed by passing them three times through a French pressure cell press at 20,000 p.s.i., and GAD activity in the supernatant fraction was determined. One unit (U) of GAD activity was defined as the amount of enzyme that produced 1 µmol of GABA in 1 min. Specific activity is expressed as U/mg of protein

### The *gadCB* operon contributes to survival under acidic conditions

**Fig. 4:**
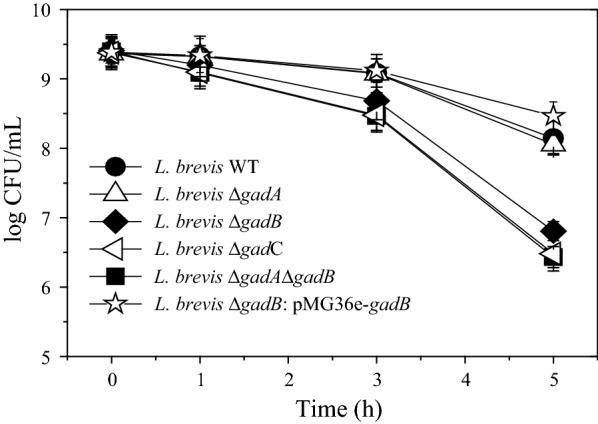
Acid resistance of *L. brevis* strains in phosphate buffer (pH 2.5) with 10 mmol/L MSG. Stationary-phase cells of *L. brevis* CGMCC1306 (WT), *L. brevis* Δ*gadA*, *L. brevis* Δ*gadB*, *L. brevis* Δ*gadC*, *L. brevis* Δ*gadA*Δ*gadB* and *L. brevis* Δ*gadB*: pMG36e-*gadB* were exposed to a pH of 2.5, and the numbers of viable cells were estimated over time. Standard deviations were calculated from the results of three independent experiments

To confirm that *gadCB* of *L. brevis* CGMCC1306 encodes proteins contributing to acid resistance, the survival of the mutants at pH 2.5 was compared with that of the wild-type strain. As shown in Fig. 4, all mutants were more sensitive to the acid challenge in the presence of MSG than the parent strain, and this distinction became more apparent over time. Similar to cell-bound GAD activity, the cell survival rate of the *L. brevis* Δ*gadA* mutant was only minimally lower after acid challenge for 5 h. However, mutant strains involving either *gadC* or *gadB* were dramatically more sensitive to acid. Additionally, this transition in the *gadC* mutant tended to be more obvious as the duration of exposure to low pH increased. Notably, the most dramatic decreases were observed with the exquisitely sensitive Δ*gadA*Δ*gadB* mutant, which showed an approximately 1.7-log reduction in survival relative to the wild-type strain after exposure to acid for 5 h. Moreover, it was also noted that the sensitivity of the Δ*gadB* complementation strain *L. brevis* Δ*gadB*: pMG36e-*gadB* was less than that of the Δ*gadB* mutant and wild-type in this phase of growth (Fig. 4), which corresponds well with the GAD activity data reported above (Table 2). In conclusion, *L. brevis* mutants deleted for *gadC* or *gadB* were sensitive to acid, which indicates that both glutamate decarboxylase and the antiporter are essential for acid resistance. Furthermore, GadB, together with GadC, confer glutamate-dependent acid resistance to this LAB, with GadA playing a minor role.

### Enhancement of *L. brevis* GAD activity by increasing expression of key GAD system genes

Upon observing that the pH-dependent GadC and GadB proteins were the greatest contributors to GABA production during the fermentation process, we hypothesized that increasing the expression of key GAD system genes may improve the overall GAD activity of *L. brevis*. Therefore, the *gadB* and *gadCB* segments were cloned into the nisin-inducible expression vector pNZ8148 [39] and subsequently transformed into *L. brevis* 9530 [28] to form *L. brevis* 9530: pNZ8148-*gadB* and *L. brevis* 9530: pNZ8148-*gadCB*, respectively. Considering the gene order of *gadB* and *gadC* in the genomes of *E. coli* [33], *S. flexneri* [34] and *B. microti* [35], another recombinant plasmid, pNZ8148-*gadBC* was also obtained and transformed into strain *L. brevis* 9530. In cells, *gadB* and *gadC* expression was under the control of the nisin-inducible *nisA* promoter (Additional file 3). After induction with 10 ng/mL nisin, samples of recombinant *L. brevis* strains grown in GYP fermentation medium under acidic conditions (pH 5.2) were collected at intervals during cultivation for extraction or determination of cell-bound GAD activity. According to SDS-PAGE analysis of whole-cell proteins, the bands observed were consistent with the predicted molecular mass of GadB (53.5 kDa) and GadC (55.2 kDa; Additional file 3), indicating that the two enzymes were well expressed under the *nisA* promoter in *L. brevis*.

**Fig. 5:**
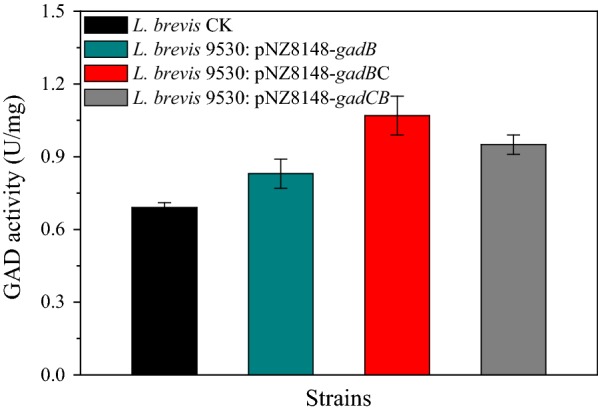
Cell-bound GAD activities of recombinant *L. brevis* strains. Cells were collected at exponential-phase (24 h). One unit (U) of GAD activity was defined as the amount of cells that produced 1 µmol/L of GABA in 1 min at pH 4.8. The specific activity of GAD was defined as U/mg DCW

To evaluate whether overexpression of GadB and GadC effectively enhanced GABA productivity, the GAD activities of these engineered *L. brevis* strains were measured. As shown in Fig. 5, after 24 h of incubation, the cell-bound GAD activities of *L. brevis* 9530: pNZ8148-*gadB*, *L. brevis* 9530: pNZ8148-*gadCB* and *L. brevis* 9530: pNZ8148-*gadBC* were 0.83 ± 0.06, 0.95 ± 0.04 and 1.07 ± 0.08 U/mg DCW, respectively. As expected, all three recombinant strains exhibited higher activities than did the wild-type strain (0.69 ± 0.02 U/mg DCW). In addition, the increased GAD activity in the recombinant *L. brevis* strain harboring pNZ9530/pNZ8148-*gadBC* system was the best compared to that of the other strains. Therefore, this strain was selected for a thorough characterization of GABA production.

### Enhanced production of GABA using a two-stage pH and temperature control with substrate feeding strategy

**Fig. 6:**
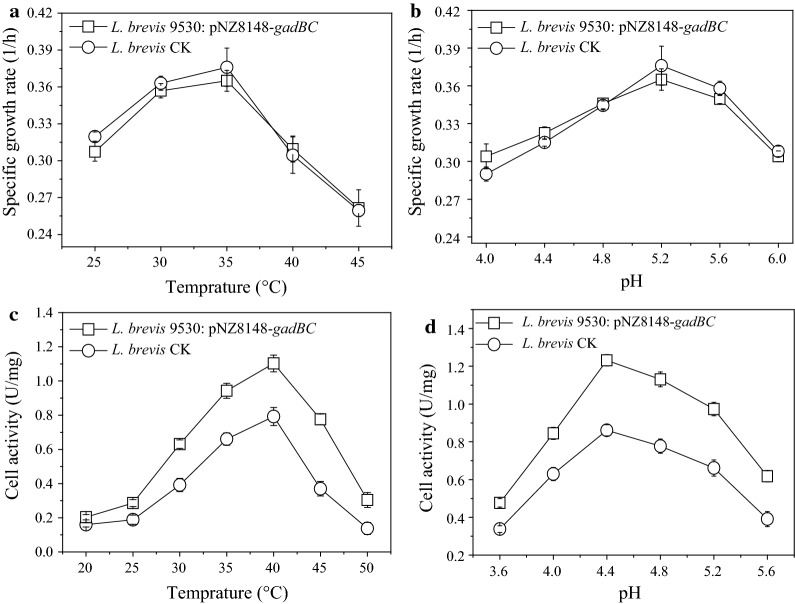
Effects of temperature (**a**) and pH (**b**) on the effective maximum specific growth rate of *Lb. brevis*. *L. brevis* CK and *L. brevis* 9530: pNZ8148-*gadBC* cells were cultured in GYP medium at different temperatures and pH values. Influence of temperature (**c**) and pH (**d**) on the cell-bound GAD activity of *L. brevis*. *L. brevis* CK and *L. brevis* 9530: pNZ8148-*gadBC* cells were grown to the late exponential growth phase (24 h) under acidic conditions (pH 5.2). GAD activities were measured in 0.2 mol/L sodium acetate buffer containing 60 mmol/L of MSG

Temperature and pH are two of the well-documented factors influencing cell growth and activity during fermentation [17, 29]. To achieve optimal conditions for maximum yield of GABA, the effects of pH and temperature on the growth and GAD activity of *L. brevis* cells were determined. At low temperatures, the specific growth rate of *L. brevis* 9530: pNZ8148-*gadBC* and *L. brevis* CK increased as temperature increased, with the maximum value observed at 35 °C, above which the specific growth rate tended to decrease (Fig. 6a). Similarly, the effect of pH on the specific growth rate is shown in Fig. 6b. The optimum pH for growth of engineered strain was pH 5.2, which was also consistent with that of *L. brevis* CK. Furthermore, the effect of temperature on cell-bound GAD activity was examined at temperatures ranging from 20 to 50 °C (Fig. 6c). At 20 °C, the GAD activity of the engineered strain (0.20 ± 0.03 U/mg DCW) was similar to that of the control strain (0.16 ± 0.02 U/mg DCW). As expected, GAD activity increased with temperature increase for both strains, but the increase was larger for *L. brevis* 9530: pNZ8148-*gadBC*, approximately 39.2% higher at the optimum temperature (40 °C) than *L. brevis* CK. Moreover, it is worth noting that the highest GAD activity of *L. brevis* 9530: pNZ8148-*gadBC* was obtained at pH 4.4, followed by pH 4.8, pH 5.2 and pH 4.0 (Fig. 6d). Obviously, the optimum pH for cell growth and GAD activity is not fully coupled, as was the case for temperature.

**Fig. 7:**
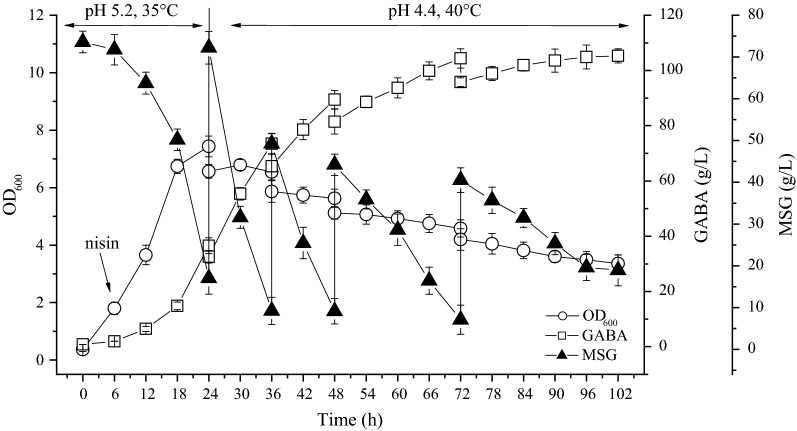
Time course of cell growth, GABA production and residual MSG during the two-stage fed-batch fermentation. *L. brevis* 9530: pNZ8148-*gadBC* was cultured in a 5-L fermentor under the following conditions: medium volume 2 L, inoculum size 10% (v/v), agitation speed 100 rpm, and fermentation time 102 h. After cultivation at pH 5.2 and 35 °C for 24 h, the culture temperature and pH were changed and maintained at 40 °C and 4.4, respectively, during the second phase (24–102 h). At 24 h, 36 h, 48 h and 72 h, 200-mL aliquots of MSG (140 g per aliquot) were supplemented into the bioreactor. The pH was maintained at the set value with the addition of 3 mol/L H_2_SO_4_ and 3 mol/L NaOH

To address the coupling of cell growth with GAD activity described above, a fed-batch fermentation with two-stage pH and temperature control strategy was proposed for improving GABA production. In the first stage (0–24 h), pH and temperature were set at pH 5.2 and 35 °C, respectively, to promote cell growth; in the second stage (24–102 h), the pH and temperature were changed to 4.4 and 40 °C, respectively, to favor GABA production. The fermentation dynamics of *L. brevis* 9530: pNZ8148-*gadBC* are shown in Fig. 7. The GABA content increased rapidly with fermentation time from 12 to 48 h after inoculation, increased slowly from 48 to 72 h, and then hardly increased as time progressed. After 102 h of fermentation, the volume of culture broth had increased to approximately 3.7 L due to the inoculation, substrate feeding and addition of neutralizer. Additionally, GABA concentrations were 89.45 ± 3.24 g/L, 104.38 ± 3.47 g/L and 105.32 ± 2.53 g/L at 48 h, 72 h and 102 h, respectively. GABA production by strain *L. brevis* CK with the proposed two-stage fed-batch strategy was also assessed (Additional file 4). However, the GABA concentrations were found to be 72.91 ± 2.46 g/L, 82.47 ± 2.49 g/L and 86.35 ± 3.71 g/L at 48 h, 72 h and 102 h, respectively, notably lower than those of the engineered strain. Based on a comprehensive consideration of GABA productivity and economic analysis, 72 h is recommended as the optimal fermentation time in future practical production.

## Discussion

Diverse mechanisms for pH sensing and cytoplasmic pH homeostasis enable most LAB to tolerate or grow at external pH values that are outside the cytoplasmic pH range that they must maintain for growth [6, 7]. Among the most preferred mechanisms are the pumping out of protons, production of alkaline compounds and proton-consuming decarboxylation reactions [7]. Nonetheless, research increasingly suggests that the acid resistance of LAB might be strain specific and stress specific because of the genetic diversity of these acid alleviating systems among different strains [12, 23]. Furthermore, each of the various components of these pathways can be adapted in several different ways to promote survival in acidic environments [7, 32]. Therefore, the challenge becomes understanding how acid stress response systems have adapted in an individual LAB species. As mentioned above, several systems have been employed to withstand low-pH stress, but the GAD system is regarded as one of the most important pathways in some LAB strains [9, 10]. Glutamate decarboxylation to GABA contributes to the acid resistance of *E. coli* [33], *Listeria monocytogenes* [40], *S. flexneri* [34], *L. lactis* [9] *and B. microti* [35] have been characterized. However, research on the physiological function and regulatory mechanism of the GAD system in *Lactobacillus* species is scarce. Given the link between acid resistance phenotypes and the organization of GAD system genes, the present study was initiated to investigate the contribution of *gad* genes to the acid tolerance of the high GABA-producing strain *L. brevis*.

*Lactobacillus brevis* CGMCC1306 contains genes encoding two GADs (GadA and GadB) and a glutamate-GABA antiporter (GadC). In *E. coli*, *S. flexneri* and *B. microti*, *gadC* genes are preceded by *gadB* [33–35], whereas *gadC* in *L. brevis* is located adjacent to but upstream of *gadB* [23]. Indeed, the arrangement of *gad* genes is a common characteristic shared by most GABA-producing LAB strains (Fig. 1b). To our knowledge, after *E. coli* and *L. monocytogenes*, *L. brevis* is only the third species in which two glutamate decarboxylase genes have been identified. Based on an analysis of the genetic organization of the GAD system, the physiological adaptation of this strain to different culture conditions was further evaluated using quantitative reverse transcription PCR. The results demonstrated that *gadCB* forms an operon in strain *L. brevis* CGMCC1306. Moreover, expression of the *gadCB* operon was greatly upregulated in response to low ambient pH when cells enter the late exponential growth phase, whereas expression of the *gadA* gene was consistently lower and did not change in response to acid or neutral conditions. There are also obvious differences in *gadR* expression levels among LAB strains. In *L. brevis* NCL912 [27], the expression levels of *gadR* were 13- to 155-fold higher than those of *gadCB* throughout the culture period, though far lower expression levels were observed for *gadR* than for *gadCB* in *L. brevis* CGMCC1306. In addition, *gadR* in *L. lactis* is constitutively expressed [9].

Although transcriptional levels are typically analyzed by measurement of relevant mRNA abundance in *L. brevis* NCL912 [27], *L. brevis* TMW 1.6 [26], *L. brevis* TMW 1.465 [26] and *L. brevis* NPS-QW-145 [23], direct experimental evidence for the detailed contribution of *gad* genes to acid tolerance and GABA production is lacking. Therefore, a number of acid-sensitive mutants were constructed to examine the regulatory mechanisms involved in acid resistance. Similar to *L. lactis* [9], *L. monocytogenes* [40] and *B. microti* [35], *L. brevis* mutants deleted for *gadC* or *gadB* were acid sensitive, indicating that both glutamate decarboxylase and the antiporter are essential for the observed acid resistance of this organism. This is also in agreement with the acid sensitivity of *E. coli* and *S. flexneri gadC* mutants [34, 41] and the *L. reuteri gadB* mutant [12] identified in previous mutagenesis studies. However, a deletion in *gadA* resulted in only a slight loss of acid resistance. Moreover, the most acid-sensitive mutant was *L. brevis* Δ*gadA*Δ*gadB*, which demonstrated an approximately 1.7-log reduction in survival relative to the wild-type strain after exposure to acid (pH 2.5) for 5 h, as was the case for *L. monocytogenes* Δ*gadA*Δ*gadB* [40]. The above data can be summarized as follows in terms of acid resistance: wild-type> Δ*gadA*> Δ*gadB*> Δ*gadC*> Δ*gadA*Δ*gadB*. This trend is true for cell-associated GAD activities, as verified in *L. monocytogenes* [40], indicating that the ability of mutants deleted for *gad* genes to survive acid stress is proportional to their ability to utilize glutamate decarboxylation to consume intracellular protons. Thus, by sensing and consuming protons, glutamate decarboxylase-mediated proton consumption and the glutamate/GABA antiporter constitute a simple molecular system that functions efficiently to protect *L. brevis* from the acid stress encountered during fermentation. Moreover, the glutamate decarboxylation activity endowed by GadB, as opposed to that of GadA, is an indispensable step in GadCB-mediated acid resistance and cell-bound GAD activity in *L. brevis* CGMCC1306.

Due to the health benefits to humans, food-fermenting LAB are regarded as the most suitable candidates for GABA production [17], and a wide range of LAB exhibit GAD activities [18]. Owing to the great potential in large-scale fermentation for the production of food-grade GABA, the biotransformation of glutamate to GABA by *L. brevis* has been extensively investigated during the last decade [17, 23]. Regardless, nearly all of the existing studies have focused on the optimal culture and transformation conditions. In this work, improving the GAD activity of *L. brevis* was achieved by increasing the expression levels of key GAD system genes. Moreover, using the proposed two-stage pH and temperature control fed-batch fermentation strategy, the GABA production by the genetically engineered strain *L. brevis* 9530: pNZ8148-*gadBC* increased continuously, reaching a high level of 104.38 ± 3.47 g/L at 72 h. Additionally, to our knowledge, the obtained GABA level is one of the highest among LAB species grown in batch or fed-batch culture.

## Conclusions

In this study, the detailed contribution of *gad* genes to acid tolerance and GABA production in *L. brevis* was determined. Experimental results show that GAD activity endowed by GadB rather than that due to GadA is an indispensable step in GadCB-mediated acid resistance and cell-bound GAD activity in *L. brevis* CGMCC1306. Moreover, enhanced production of GABA by an engineered *L. brevis* strain was achieved using a two-stage pH and temperature control fed-batch fermentation strategy. The obtained level of GABA is one of the highest among LAB species grown in batch or fed-batch culture.

## Methods

### Bacterial strains, plasmids, and growth conditions

The bacterial strains and plasmids used in this work are listed in Table 3. *E. coli* was grown aerobically with shaking at 250 rpm in Luria‒Bertani (LB) broth at 28 or 37 °C. *L. lactis* MG1363 was grown at 28 °C in M17 medium supplemented with 0.5% glucose (GM17). *L. brevis* CGMCC1306 was generally cultured in MRS medium at 28 or 37 °C without agitation. Solid media for plating were prepared by adding 1.5% agar to the appropriate liquid media. Selective medium contained 5, 7 or 200 μg/mL erythromycin for selection of *L. lactis*, *L. brevis* or *E. coli*, respectively. Similarly, medium containing 10 or 20 μg/mL chloramphenicol was used for the selection of *L. brevis* or *E. coli*, respectively.

**Table 3:** Bacterial strains and plasmids

Strain/plasmid	Characteristics	Source/references
Strains		
*E. coli* DH5α	Transformation host; F-*φ*80 *lac*ZΔM15 Δ(*lacZYA*-*argF*) U169 *rec*A1 *end*A1 *hsd*R17 (rk−, mk+) *pho*A *sup*E44 λ- *thi*-1 *gyr*A96 *rel*A1	Invitrogen
*E. coli* MC1061	Transformation host; λ FΔ(*ara*A-*leu*)*7697 ara*D139 Δ(*codB*-*lac*)*3*=Δ*lac*74 *gal*K16 *gal*E15 *mcr*A0 *rel*A1 *rps*L150 *spo*T1 *mcr*B9999 *hsd*R2 (Str^r^)	Mobitec
*E. coli* XL1-Blue	Transformation host; *rec*A1 *end*A1 *gyr*A96 *thi*-1 *hsd*R17 *sup*E44 *rel*A1 *lac*[F′ *pro*AB *lac*IqZ∆M15 Tn10 Tet^r^]	Stratagene
*L. lactis* MG1363: pGhost4	*L. lactis* MG1363 harboring a temperature sensitive plasmid-pGhost4	[42]
*L. brevis* CGMCC 1306	Wild-type strain, originally isolated from raw milk	
*L. brevis* Δ*gadA*	*L. brevis* CGMCC1306 derivative with *gadA* deleted	This work
*L. brevis* Δ*gadB*	*L. brevis* CGMCC1306 derivative with *gadB* deleted	This work
*L. brevis* Δ*gadC*	*L. brevis* CGMCC1306 derivative with *gadC* deleted	This work
*L. brevis* Δ*gadA*Δ*gadB*	*L. brevis* CGMCC1306 derivative with both *gadA* and *gadB* deleted	This work
*L. brevis* Δ*gadB*: pMG36e-*gadB*	*L. brevis* Δ*gadB* harboring pMG36e-*gadB*	This work
*L. brevis* 9530	*L. brevis* CGMCC1306 harboring the NICE system helper plasmid pNZ9530	[28]
*L. brevis* CK	*L. brevis* CGMCC1306 harboring pNZ9530 and pNZ8148	[28]
*L. brevis* 9530: pNZ8148-*gadB*	*L. brevis* 9530 harboring pNZ8148-*gadB*	This work
*L. brevis* 9530: pNZ8148-*gadCB*	*L. brevis* 9530 harboring pNZ8148-*gadCB*	This work
*L. brevis* 9530: pNZ8148-*gadBC*	*L. brevis* 9530 harboring pNZ8148-*gadBC*	This work
Plasmids		
pNZ8148	Cm^r^, carries the nisin-inducible promoter P_*nisA*_; 3.2 kb	Mobitec
pMG36e	Em^r^, constitutive expression vector with P_32_ promoter; 3.6 kb	[43]
pMG36e-*gadB*	Em^r^, *gadB* gene was cloned into pMG36e; 5.0 kb	This work
pNZ8148-*gadCB*	Cm^r^, *gadCB* segment was cloned into pNZ8148; 6.1 kb	This work
pNZ8148-*gadB*	Cm^r^, *gadB* segment was cloned into pNZ8148; 4.6 kb	This work
pNZ8148-*gadBC*	Cm^r^, *gadBC* segment was cloned into pNZ8148; 6.1 kb	This work
pGhost4	Em^r^, integration vector, thermosensitive replicative plasmid in LAB, derivative of pGK12; 3.8 kb	[42]

### Fermentation experiments

During batch and fed-batch fermentations, *L. brevis* cells were grown in glucose yeast extract peptone (GYP) medium as described previously [29], with minor modifications (g/L): glucose, 20; yeast extract, 15; peptone, 5; MSG, 75; CH_3_COONa, 3; FeSO_4_·7H_2_O, 0.001; MgSO_4_·7H_2_O, 0.03; NaCl, 0.001; MnSO_4_·4H_2_O, 0.02. In 48 h batch fermentations, temperature was maintained at 35 °C, and the pH was kept constant at 5.2 or 6.8. To enhance GABA production, a two-stage pH and temperature control with substrate-feeding strategy was developed. The experiments were performed in a 5-L fermentor under the following conditions: medium volume 2 L, inoculum size 10% (v/v), agitation speed 100 rpm, and fermentation time 102 h. In particular, after cultivation at pH 5.2 and 35 °C for 24 h, the culture temperature and pH were changed and maintained at 40 °C and pH 4.4, respectively, during the second phase (24–102 h). Additionally, 200-mL aliquots of MSG (140 g per aliquot) were supplemented into the bioreactor at 24 h, 36 h, 48 h and 72 h, respectively. The pH was maintained at the set value with the addition of 3 mol/L H_2_SO_4_ or 3 mol/L NaOH.

### Amino acid comparison of GAD system genes and gene loci in LAB

The *gadR*, *gadC*, *gadB*, and *gadA* gene sequences of *L. brevis* ATCC367 (GenBank: CP000416) were used to identify homologous genes encoding *gadR, gadC, gadB* and *gadA* in the genome of *L. brevis* CGMCC1306. Gene loci of putative GAD system genes in *L. buchneri* NRRL B-30929, *L. sakei* A156, *L. brevis* CGMCC1306, *L. reuteri* 100-23, *L. plantarum* WCFS1, *L. acidophilus* NCFM, *L. lactis* subsp. *lactis* CV56 and *L. lactis* subsp. *cremoris* MG1363 were analyzed with the BLASTx program against NCBI databases (http://blast.ncbi.nlm.nih.gov/Blast.cgi). The sequences of GAD system proteins from various species were retrieved from NCBI and aligned to calculate similarity scores. Gene or protein sequences were aligned using ClustalX, and a phylogenetic tree was constructed with the neighbor-joining (NJ) method using MEGA 4.0. The *gad* genes in *L. brevis* CGMCC1306 were cloned and sequenced. The sequences of the *16S rDNA*, *gadR*, *gadCB* operon and *gadA* were deposited in the GenBank database under accession numbers HQ726794, KU759571, JQ246952 and GU987102.1, respectively.

### Generation and verification of deletion mutants

The chromosomal *gadA* gene was deleted in *L. brevis* CGMCC1306 as follows. DNA fragments of 735 bp and 682 bp carrying the upstream and downstream regions of the *gadA* gene, respectively, were generated by PCR. These fragments contained *Kpn*I/*Pst*I and *Pst*I/*Xba*I restriction sites at their 5′ and 3′ ends, respectively, which were introduced using *gadA* upstream and downstream primers (Additional file 5). The PCR-amplified products were digested with the corresponding restriction endonucleases, ligated, and cloned into the pGhost4 vector [37, 42] and its derivative to generate pGh4-Δ*gadA*-*U* and pGh4-Δ*gadA*-*U*-*D*, respectively. *E. coli* XL1-Blue cells were used as the intermediate host. The absence of PCR-induced mutations in the insert corresponding to the fused upstream and downstream regions of the *gadA* gene was verified by sequencing. The recombinant plasmid pGh4-Δ*gadA*-*U*-*D* was transformed into *L. brevis* by electroporation as described in our previous work [28]. The transformant was selected on MRS plates containing erythromycin at 28 °C. After homologous recombination into the chromosome, and clearing of the plasmid as previously described [37], the deletion was confirmed by colony PCR amplification using the flanking primers *up* F and *down* R. Similarly, *L. brevis* strains Δ*gadB* and Δ*gadC* were constructed, and the Δ*gadA*Δ*gadB* double mutant was further prepared using strain Δ*gadA* as the template for homologous recombination of Δ*gadB*.

### Construction of plasmids and strains overexpressing key GAD system genes

For overexpression of *gad* genes in *L. brevis*, *gadC, gadB* and *gadCB* segments were amplified from the genomic DNA of strain *L. brevis* CGMCC1306. Information regarding the primers is listed in Additional file 6. The *gadCB* segment was amplified using the *gadCB*-F and *gadCB*-R primer pair and ligated into the *Pst*I and *Hin*dIII restriction sites of the vector pNZ8148 [39] to produce pNZ8148-*gadCB*. Similarly, the recombinant plasmids pNZ8148-*gadB* and pNZ8148-*gadBC* were obtained successively. *E. coli* MC1061 was used as the intermediate host and transformed with these constructs. The recombinant plasmids were confirmed by restriction enzyme analysis and DNA sequencing, and then transformed into *L. brevis* 9530 [28] (*L. brevis* CGMCC1306 harboring the facilitator plasmid pNZ9530) by electroporation. For expression of *gadB* in *L. brevis* Δ*gadB*, the *gadB* segment was amplified with primers *gadB*-F2 and *gadB*-R2, digested with *Sal*I and *Hin*dIII, and then ligated into the constitutive expression vector pMG36e [43]. *E. coli* DH5α was used as the intermediate host. The recombinant plasmid was also transformed into strain *L. brevis* Δ*gadB* to form *L. brevis* Δ*gadB*: pMG36e-*gadB*.

### Quantitative real-time PCR of *gad* genes

The growth of *L. brevis* in GYP medium was monitored by measuring optical density (OD) at 600 nm. Cells at different growth phases were harvested, and total RNA, stabilized with RNA protect Bacteria Reagent (Qiagen), was isolated in triplicate using RNeasy Mini Kit (Qiagen) according to the manufacturer’s protocol. RNA quantification and quality assessment were carried out by UV spectrometry (OneDrop, OD-1000). For quantitative real-time PCR (qRT-PCR), extracted mRNAs were reverse transcribed to complementary DNA (cDNA) using the ReverTra Ace^®^ qPCR RT kit (Toyobo Co., Ltd., Japan) and quantified via real-time PCR using SYBR^®^ Premix Ex TaqTM II (Takara). Relative expression of target genes was calculated according to the comparative 2^−ΔΔCt^ method described by Livak and Schmittgen [44]. The *16S rRNA* gene was used as a housekeeping gene, and a no-template control was used as a negative control. Ct values were normalized to the samples cultured for 6 h (control group). The primers used for housekeeping and *gad* genes are listed in Additional file 7. All quantitative PCRs were performed in triplicate with a StepOnePlus™ Real-Time PCR System.

### Survival under acidic conditions

To evaluate acid resistance, early stationary phase cells were obtained from cultures grown until the OD stopped increasing exponentially. The cells were then washed in 50 mmol/L potassium phosphate buffer (pH 7.0), and resuspended in 50 mmol/L potassium phosphate buffer (pH 2.5) to an OD_600_ of 1.0. A pH of 2.5 adjusted with HCl was chosen to match the conditions previously used to determine the effect of glutamate decarboxylase on acid resistance in *L. reuteri* [45]. Samples were collected after 0, 1, 3, and 5 h of incubation at pH 2.5 to monitor bacterial survival. For determination of bacterial survival, the samples were immediately mixed with phosphate-buffered saline (PBS, 137 mmol/L NaCl, 2.7 mmol/L KCl, 10 mmol/L Na_2_HPO_4_, and 2 mmol/L KH_2_PO_4_, pH 7.4) and diluted in PBS prior to plating on MRS agar medium. The plates were incubated at 37 °C for 48 h, and colonies were counted to assess survival under lethal acidic conditions. Where indicated, 10 mmol/L of MSG was added.

### GAD activity assay

Cell-bound GAD activity was determined by measuring the amount of GABA formed at 37 °C in a reaction mixture containing 0.1 mg (dry cell weight)/mL cell biomass, 0.2 mol/L sodiumacetate buffer (pH 4.8), and 60 mmol/L MSG. One unit (U) of GAD activity was defined as the amount of cells that produced 1 µmol of GABA in 1 min under the above conditions. Specific activity was defined as U/mg DCW cells. The cytoplasmic GAD activity of *L. brevis* was also determined. Cells were collected and lysed by passage three times through a French pressure cell press (Constant Cell Disruption Systems, UK) at 20,000 p.s.i., followed by centrifugation of the homogenate to remove cellular debris (10,000×*g*, 4 °C, 10 min). The GAD activity of the supernatant fraction was determined. One unit (U) of GAD activity was defined as the amount of enzyme that produced 1 µmol of GABA in 1 min, and specific activity is expressed as U/mg of protein. Concentrations of Glu and GABA were determined by reversed-phase high-performance liquid chromatography (RP-HPLC), as described by Marquez et al. [46].

## Additional files

**Additional file 1.** **Figure S1.** (A), Gene organization of the *gad* gene cluster in *L. brevis* CGMCC1306 (not drawn to scale). (B), Nucleotide sequence and expression signals of the *gad* gene cluster in *L. brevis* CGMCC1306. Parts of deduced amino acid sequences of the genes are given below the sequence. Facing arrows, inverted repeats; -10 and -35 promoter sequences are underlined and in boldface; vertical arrows, transcription stare points; rbs, ribosome-binding site (nucleotides indicated in lower case). Stop codons are indicated with asterisks; start codons are in bold face.

**Additional file 2.** **Figure S2.** (A), Transcription of *gadR* is independent from the *gadCB* operon. PCR results using *gad*-F_1_ and *gad*-R_1_ as primers. The expected band (~ 573 bp) was only visible in the lane C. M: DNA marker; A: cDNA as the template. B: mRNA as the template. C: genome DNA as the template. *gad*-F_1_: GTCAAACAACAATTGGCATC, *gad*-R_1_: CAGCCGATAATGAAATACATC. (B), The *gadB* is co-transcribed in tandem with the upstream *gadC* gene. PCR results using *gad*-F_2_ and *gad*-R_2_ as primers. The expected band (~ 571 bp) was visible only in the lane A. M: DNA marker; A: cDNA as the template B: mRNA as the template. *gad*-F_2_: TATCTTGTACCGTTTCCACG, *gad-*R_2_: ATACATCCTTCAGAAGAACC.

**Additional file 3.** **Figure S3.** Nisin-induced overexpression of GadB and GadC in *L. brevis*. (A): The nisin-controlled gene expression system consists of two compatible replicons: an essential helper plasmid, pNZ9530, encoding the *nisRK* regulatory genes, and the expression plasmid pNZ8148, bearing the *nisA* promoter (P_*nisA*_). The *gadB*, *gadBC* or *gadCB* segments were under control of the nisin-inducible P_*nisA*_. (B): SDS-PAGE analysis of overexpression of GAD proteins. Cells were harvested after 6 hours of induction. Lane M, protein size markers (kDa). Lane 1, *L. brevis* 9530: pNZ8148-*gadCB*; Lane 2, *L. brevis* 9530: pNZ8148-*gadBC*; Lane 3, *L. brevis* 9530: pNZ8148-*gadB*; Lane 4, *L. brevis* CK.

**Additional file 4.** **Figure S4.** Time courses of cell growth, GABA production and residual MSG during the two-stage fed-batch fermentation. The *L. brevis* CK was cultured in a 5-L fermentor under the following conditions: medium volume 2 L, inoculum size 10% (v/v), agitation speed 100 rpm, and fermentation time 102 h. After cultivation at pH 5.2 and 35 °C for 24 h, the culture temperature and pH were changed and maintained at 40 °C and 4.4, respectively, during the second phase (24–102 h). At 24 h, 36 h, 48 h and 72 h, 200-mL aliquots of MSG (140 g per aliquot) were supplemented into the bioreactor. The pH was maintained at the set value with the addition of 3 mol/L H_2_SO_4_ and 3 mol/L NaOH.

**Additional file 5.** **Table S1.** Primers used for PCR amplification.

**Additional file 6.** **Table S2.** Primers used for key GAD system genes amplification.

**Additional file 7.** **Table S3.** Primer used in the quantitative PCR.

## Abbreviations

*L. brevis*: *Lactobacillus brevis*
GAD: glutamate decarboxylase
GABA: γ-aminobutyric acid
MSG: *L*-monosodium glutamate
GRAS: generally recognized as safe
DCW: mg dry cell weight
PCR: polymerase chain reaction
NCBI: National Center for Biotechnology Information
RP-HPLC: reversed phase high-performance liquid chromatography
SDS-PAGE: sodium dodecyl sulfate polyacrylamide gel electrophoresis

## References

[1] Gaspar P, Carvalho AL, Vinga S, Santos H, Neves AR (2013). From physiology to systems metabolic engineering for the production of biochemicals by lactic acid bacteria. Biotechnol Adv.

[2] Heller KJ (2001). Probiotic bacteria in fermented foods: product characteristics and starter organisms. Am J Clin Nutr.

[3] Cotter PD, Hill C (2003). Surviving the acid test: responses of gram-positive bacteria to low pH. Microbiol Mol Biol Rev.

[4] Schiffrin EJ, Blum S (2001). Food processing: probiotic microorganisms for beneficial foods. Curr Opin Biotechnol.

[5] Quivey RG, Kuhnert WL, Hahn K (2000). Adaptation of oral streptococci to low pH. Adv Microb Physiol.

[6] Krulwich TA, Sachs G, Padan E (2011). Molecular aspects of bacterial pH sensing and homeostasis. Nat Rev Microbiol.

[7] Lund P, Tramonti A, De Biase D (2014). Coping with low pH: molecular strategies in neutralophilic bacteria. FEMS Microbiol Rev.

[8] van de Guchte M, Serror P, Chervaux C, Smokvina T, Ehrlich SD, Maguin E (2002). Stress responses in lactic acid bacteria. Antonie Van Leeuwenhoek.

[9] Sanders JW, Leenhouts K, Burghoorn J, Brands JR, Venema G, Kok J (1998). A chloride-inducible acid resistance mechanism in *Lactococcus lactis* and its regulation. Mol Microbiol.

[10] Feehily C, Karatzas KA (2013). Role of glutamate metabolism in bacterial responses towards acid and other stresses. J Appl Microbiol.

[11] Lagerborg VA, Clapper WE (1952). Amino acid decarboxylases of lactic acid bacteria. J Bacteriol.

[12] Su MS, Schlicht S, Ganzle MG (2011). Contribution of glutamate decarboxylase in *Lactobacillus reuteri* to acid resistance and persistence in sourdough fermentation. Microb Cell Fact.

[13] Higuchi T, Hayashi H, Abe K (1997). Exchange of glutamate and gamma-aminobutyrate in a *Lactobacillus* strain. J Bacteriol.

[14] Savage K, Firth J, Stough C, Sarris J (2018). GABA-modulating phytomedicines for anxiety: a systematic review of preclinical and clinical evidence. P hytother Res.

[15] Nemeroff CB (2003). The role of GABA in the pathophysiology and treatment of anxiety disorders. Psychopharmacol Bull.

[16] He S, Zhang Y, Wang D, Tao K, Zhang S, Wei L, Chen Q (2016). Rapamycin/GABA combination treatment ameliorates diabetes in NOD mice. Mol Immunol.

[17] Li H, Cao Y (2010). Lactic acid bacterial cell factories for gamma-aminobutyric acid. Amino Acids.

[18] Dhakal R, Bajpai VK, Baek KH (2012). Production of GABA (gamma-aminobutyric acid) by microorganisms: a review. Braz J Microbiol.

[19] Wu Q, Shah NP (2017). High gamma-aminobutyric acid production from lactic acid bacteria: emphasis on *Lactobacillus brevis* as a functional dairy starter. Crit Rev Food Sci Nutr.

[20] Qiu T, Huang GD, Cao YS (2010). Production of gamma-aminobutyric acid by *Lactobacillus brevis* NCL912 using fed-batch fermentation. Microb Cell Fact.

[21] Lyu CJ, Zhao WR, Hu S, Huang J, Lu T, Jin ZH, Mei LH, Yao SJ (2017). Physiology oriented engineering strategy to improve gamma-aminobutyrate production in *Lactobacillus brevis*. J Agric Food Chem.

[22] Wu Q, Law YS, Shah NP (2015). Dairy *Streptococcus thermophilus* improves cell viability of *Lactobacillus brevis* NPS-QW-145 and its gamma-aminobutyric acid biosynthesis ability in milk. Sci Rep.

[23] Wu Q, Tun HM, Law YS, Khafipour E, Shah NP (2017). Common distribution of *gad* operon in *Lactobacillus brevis* and its GadA contributes to efficient GABA synthesis toward cytosolic near-neutral pH. Front Microbiol.

[24] Wu Q, Shah NP (2018). Restoration of GABA production machinery in *Lactobacillus brevis* by accessible carbohydrates, anaerobiosis and early acidification. Food Microbiol.

[25] Yunes RA, Poluektova EU, Dyachkova MS, Klimina KM, Kovtun AS, Averina OV, Orlova VS, Danilenko VN (2016). GABA production and structure of *gadB/gadC* genes in *Lactobacillu*s and *Bifidobacterium* strains from human microbiota. Anaerobe.

[26] Schurr BC, Behr J, Vogel RF (2013). Role of the GAD system in hop tolerance of *Lactobacillus brevis*. Eur Food Res Technol.

[27] Li H, Li W, Liu X, Cao Y (2013). gadA gene locus in *Lactobacillus brevis* NCL912 and its expression during fed-batch fermentation. FEMS Microbiol Lett.

[28] Lyu C, Hu S, Huang J, Luo M, Lu T, Mei L, Yao S (2016). Contribution of the activated catalase to oxidative stress resistance and gamma-aminobutyric acid production in *Lactobacillus brevis*. Int J Food Microbiol.

[29] Huang J, Mei LH, Xia J (2007). Application of artificial neural network coupling particle swarm optimization algorithm to biocatalytic production of GABA. Biotechnol Bioeng.

[30] de Ruyter PG, Kuipers OP, Beerthuyzen MM, van Alen-Boerrigter I, de Vos WM (1996). Functional analysis of promoters in the nisin gene cluster of *Lactococcus lactis*. J Bacteriol.

[31] Azcarate-Peril MA, Altermann E, Hoover-Fitzula RL, Cano RJ, Klaenhammer TR (2004). Identification and inactivation of genetic loci involved with *Lactobacillus acidophilus* acid tolerance. Appl Environ Microbiol.

[32] Teixeira JS, Seeras A, Sanchez-Maldonado AF, Zhang C, Su MS, Ganzle MG (2014). Glutamine, glutamate, and arginine-based acid resistance in *Lactobacillus reuteri*. Food Microbiol.

[33] De Biase D, Pennacchietti E (2012). Glutamate decarboxylase-dependent acid resistance in orally acquired bacteria: function, distribution and biomedical implications of the *gadBC* operon. Mol Microbiol.

[34] Waterman SR, Small PL (1996). Identification of σ^s^-dependent genes associated with the stationary-phase acid-resistance phenotype of *Shigella flexneri*. Mol Microbiol.

[35] Grassini G, Pennacchietti E, Cappadocio F, Occhialini A, De Biase D (2015). Biochemical and spectroscopic properties of *Brucella microti* glutamate decarboxylase, a key component of the glutamate-dependent acid resistance system. FEBS Open Bio.

[36] Sa HD, Park JY, Jeong SJ, Lee KW, Kim JH (2015). Characterization of glutamate decarboxylase (GAD) from *Lactobacillus sakei* A156 isolated from jeot-gal. J Microbiol Biotechnol.

[37] Maguin E, Prevost H, Ehrlich SD, Gruss A (1996). Efficient insertional mutagenesis in lactococci and other gram-positive bacteria. J Bacteriol.

[38] Ma D, Lu P, Yan C, Fan C, Yin P, Wang J, Shi Y (2012). Structure and mechanism of a glutamate-GABA antiporter. Nature.

[39] Mierau I, Kleerebezem M (2005). 10 years of the nisin-controlled gene expression system (NICE) in *Lactococcus lactis*. Appl Microbiol Biotechnol.

[40] Cotter PD, Gahan CG, Hill C (2001). A glutamate decarboxylase system protects *Listeria monocytogenes* in gastric fluid. Mol Microbiol.

[41] Hersh BM, Farooq FT, Barstad DN, Blankenhorn DL, Slonczewski JL (1996). A glutamate-dependent acid resistance gene in *Escherichia coli*. J Bacteriol.

[42] Maguin E, Duwat P, Hege T, Ehrlich D, Gruss A (1992). New thermosensitive plasmid for gram-positive bacteria. J Bacteriol.

[43] van de Guchte M, van der Vossen JM, Kok J, Venema G (1989). Construction of a lactococcal expression vector: expression of hen egg white lysozyme in *Lactococcus lactis* subsp. *lactis*. Appl Environ Microbiol.

[44] Livak KJ, Schmittgen TD (2001). Analysis of relative gene expression data using real-time quantitative PCR and the 2^−ΔΔCT^ method. Methods.

[45] Su MS, Schlicht S, Ganzle MG (2011). Contribution of glutamate decarboxylase in *Lactobacillus reuteri* to acid resistance and persistence in sourdough fermentation. Microb Cell Fact.

[46] Marquez FJ, Quesada AR, Sanchezjimenez F, Decastro IN (1986). Determination of 27 Dansyl amino acid derivatives in biological fluids by reversed phase high performance liquid chromatography. J Chromatogr.

