# Supplementary material for: Exploring the contributions of two glutamate decarboxylase isozymes in Lactobacillus brevis to acid resistance and γ-aminobutyric acid production
Source: Microb Cell Fact. 2018 Nov 19;17:180. doi: 10.1186/s12934-018-1029-1 (PMC6240960; doi:10.1186/s12934-018-1029-1)

**Additional file 1**

**Figure S1.** (A), Gene organization of the *gad* gene cluster in *L. brevis* CGMCC1306 (not drawn to scale). Putative intrinsic terminators (
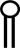
) and their calculated free energy are indicated. (B), Nucleotide sequence and expression signals of the *gad* gene cluster in *L. brevis* CGMCC1306. Parts of deduced amino acid sequences of the genes are given below the sequence. Facing arrows, inverted repeats; -10 and -35 promoter sequences are underlined and in boldface; vertical arrows, transcription stare points; rbs, ribosome-binding site (nucleotides indicated in lower case). Stop codons are indicated with asterisks; start codons are in bold face.


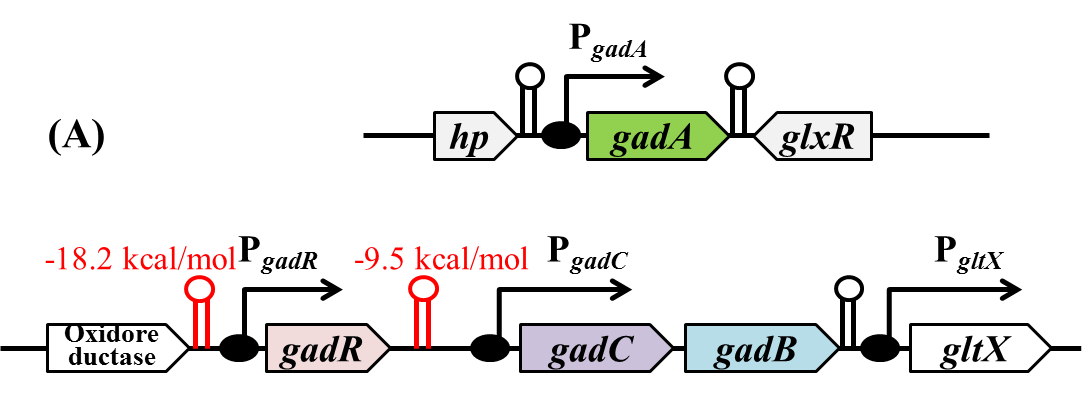


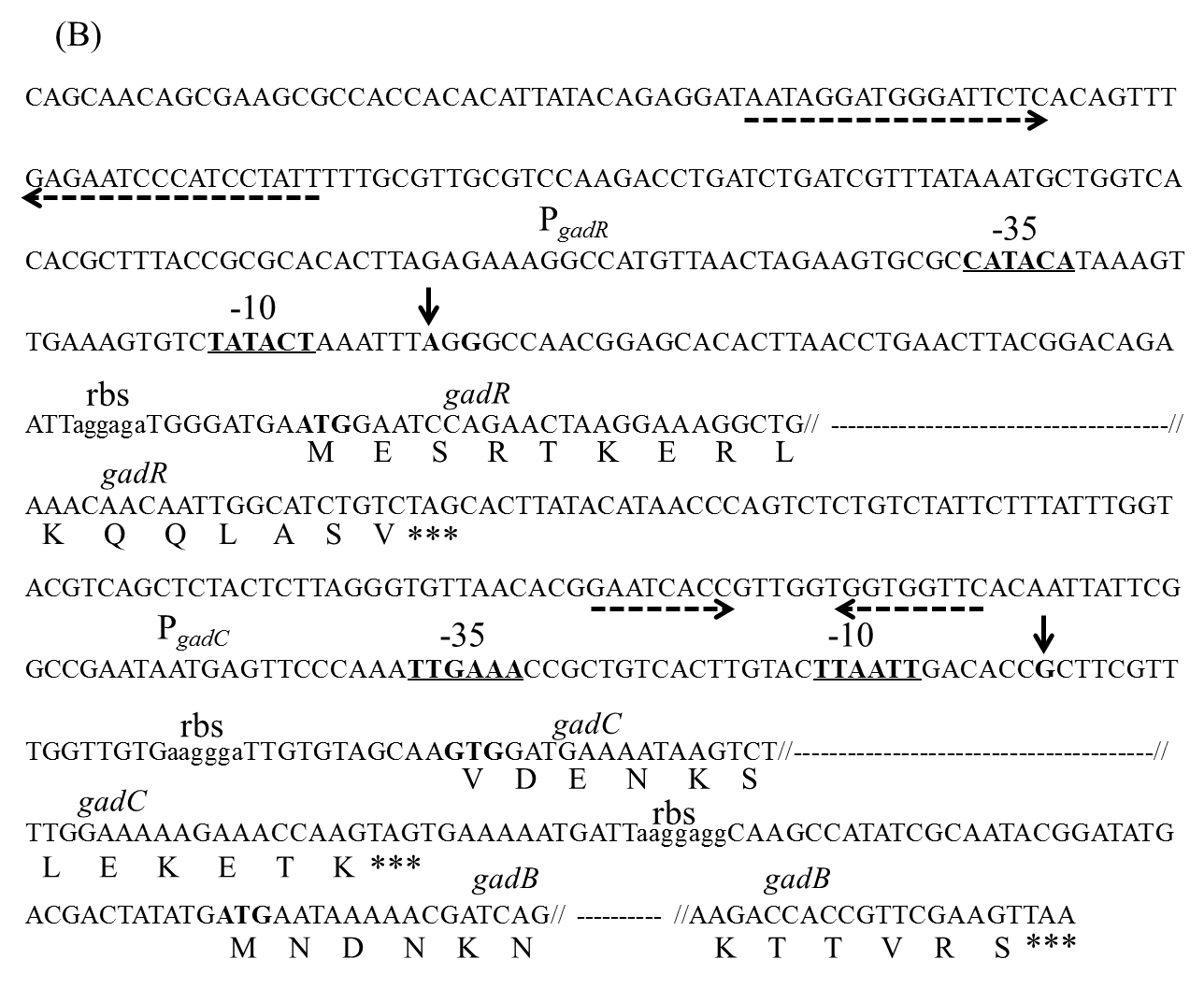

Supplement: Supplementary file 1 — Additional file 1. Figure S1. (A), Gene organization of the gad gene cluster in L. brevis CGMCC1306 (not drawn to scale). (B), Nucleotide sequence and expression signals of the gad gene cluster in L. brevis CGMCC1306. Parts of deduced amino acid sequences of the genes are given below the sequence. Facing arrows, inverted repeats; -10 and -35 promoter sequences are underlined and in boldface; vertical arrows, transcription stare points; rbs, ribosome-binding site (nucleotides indicated in lower case). Stop codons are indicated with asterisks; start codons are in bold face. [file 12934_2018_1029_MOESM1_ESM.docx]
