# Supplementary material for: Exploring the contributions of two glutamate decarboxylase isozymes in Lactobacillus brevis to acid resistance and γ-aminobutyric acid production
Source: Microb Cell Fact. 2018 Nov 19;17:180. doi: 10.1186/s12934-018-1029-1 (PMC6240960; doi:10.1186/s12934-018-1029-1)

**Additional file 2**

**Figure S2.** (A), Transcription of *gadR* is independent from the *gadCB* operon. PCR results using *gad*-F_1_ and *gad*-R_1_ as primers. The expected band (~573 bp) was only visible in the lane C. M: DNA marker; A: cDNA as the template. B: mRNA as the template. C: genome DNA as the template. *gad*-F_1_: GTCAAACAACAATTGGCATC, *gad*-R_1_: CAGCCGATAATGAAATACATC.


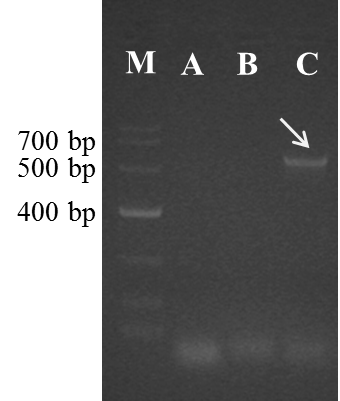


**Figure S2.** (B), The *gadB* is co-transcribed in tandem with the upstream *gadC* gene. PCR results using *gad*-F_2_ and *gad*-R_2_ as primers. The expected band (~571 bp) was visible only in the lane A. M: DNA marker; A: cDNA as the template B: mRNA as the template. *gad*-F_2_: TATCTTGTACCGTTTCCACG, *gad-*R_2_: ATACATCCTTCAGAAGAACC.


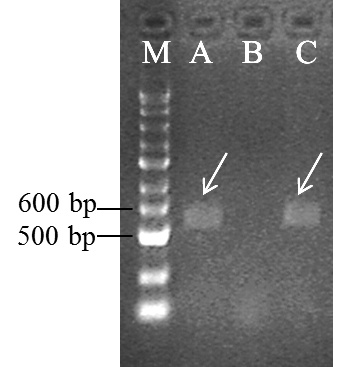

Supplement: Supplementary file 2 — Additional file 2. Figure S2. (A), Transcription of gadR is independent from the gadCB operon. PCR results using gad-F1 and gad-R1 as primers. The expected band (~ 573 bp) was only visible in the lane C. M: DNA marker; A: cDNA as the template. B: mRNA as the template. C: genome DNA as the template. gad-F1: GTCAAACAACAATTGGCATC, gad-R1: CAGCCGATAATGAAATACATC. (B), The gadB is co-transcribed in tandem with the upstream gadC gene. PCR results using gad-F2 and gad-R2 as primers. The expected band (~ 571 bp) was visible only in the lane A. M: DNA marker; A: cDNA as the template B: mRNA as the template. gad-F2: TATCTTGTACCGTTTCCACG, gad-R2: ATACATCCTTCAGAAGAACC. [file 12934_2018_1029_MOESM2_ESM.docx]
