# Supplementary material for: Exploring the contributions of two glutamate decarboxylase isozymes in Lactobacillus brevis to acid resistance and γ-aminobutyric acid production
Source: Microb Cell Fact. 2018 Nov 19;17:180. doi: 10.1186/s12934-018-1029-1 (PMC6240960; doi:10.1186/s12934-018-1029-1)

**Additional file 3**

**Figure S3.** Nisin-induced overexpression of GadB and GadC in *L. brevis*. (A): The nisin-controlled gene expression system consists of two compatible replicons: an essential helper plasmid, pNZ9530, encoding the *nisRK* regulatory genes, and the expression plasmid pNZ8148, bearing the *nisA* promoter (P*_nisA_*). The *gadB*, *gadBC* or *gadCB* segments were under control of the nisin-inducible P*_nisA_*. (B): SDS-PAGE analysis of overexpression of GAD proteins. Cells were harvested after 6 hours of induction. Lane M, protein size markers (kDa). Lane 1, *L. brevis* 9530: pNZ8148-*gadCB*; Lane 2, *L. brevis* 9530: pNZ8148-*gadBC*; Lane 3, *L. brevis* 9530: pNZ8148-*gadB*; Lane 4, *L. brevis* CK.


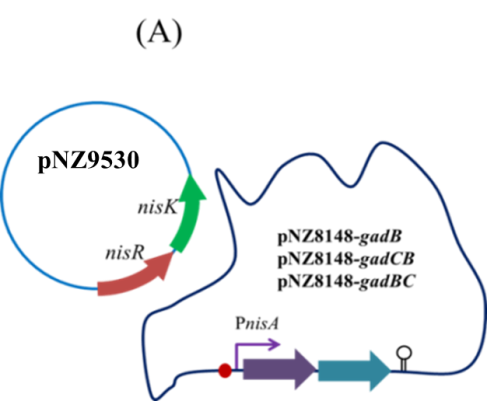

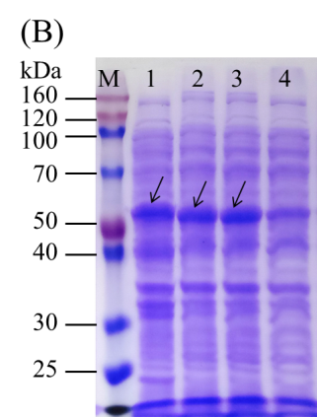

Supplement: Supplementary file 3 — Additional file 3. Figure S3. Nisin-induced overexpression of GadB and GadC in L. brevis. (A): The nisin-controlled gene expression system consists of two compatible replicons: an essential helper plasmid, pNZ9530, encoding the nisRK regulatory genes, and the expression plasmid pNZ8148, bearing the nisA promoter (PnisA). The gadB, gadBC or gadCB segments were under control of the nisin-inducible PnisA. (B): SDS-PAGE analysis of overexpression of GAD proteins. Cells were harvested after 6 hours of induction. Lane M, protein size markers (kDa). Lane 1, L. brevis 9530: pNZ8148-gadCB; Lane 2, L. brevis 9530: pNZ8148-gadBC; Lane 3, L. brevis 9530: pNZ8148-gadB; Lane 4, L. brevis CK. [file 12934_2018_1029_MOESM3_ESM.docx]
