# Supplementary material for: Exploring the contributions of two glutamate decarboxylase isozymes in Lactobacillus brevis to acid resistance and γ-aminobutyric acid production
Source: Microb Cell Fact. 2018 Nov 19;17:180. doi: 10.1186/s12934-018-1029-1 (PMC6240960; doi:10.1186/s12934-018-1029-1)
Supplement: Supplementary file 4 — Additional file 4. Figure S4. Time courses of cell growth, GABA production and residual MSG during the two-stage fed-batch fermentation. The L. brevis CK was cultured in a 5-L fermentor under the following conditions: medium volume 2 L, inoculum size 10% (v/v), agitation speed 100 rpm, and fermentation time 102 h. After cultivation at pH 5.2 and 35 °C for 24 h, the culture temperature and pH were changed and maintained at 40 °C and 4.4, respectively, during the second phase (24–102 h). At 24 h, 36 h, 48 h and 72 h, 200-mL aliquots of MSG (140 g per aliquot) were supplemented into the bioreactor. The pH was maintained at the set value with the addition of 3 mol/L H2SO4 and 3 mol/L NaOH. [file 12934_2018_1029_MOESM4_ESM.docx]

**Additional file 4**

**Fig. S4.** Time courses of cell growth, GABA production and residual MSG during the two-stage fed-batch fermentation. The *L. brevis* CK was cultured in a 5-L fermentor under the following conditions: medium volume 2 L, inoculum size 10% (v/v), agitation speed 100 rpm, and fermentation time 102 h. After cultivation at pH 5.2 and 35°C for 24 h, the culture temperature and pH were changed and maintained at 40°C and 4.4, respectively, during the second phase (24-102 h). At 24 h, 36 h, 48 h and 72 h, 200-mL aliquots of MSG (140 g per aliquot) were supplemented into the bioreactor. The pH was maintained at the set value with the addition of 3 mol/L H_2_SO_4_ and 3 mol/L NaOH.
