# Supplementary material for: Exploring the contributions of two glutamate decarboxylase isozymes in Lactobacillus brevis to acid resistance and γ-aminobutyric acid production
Source: Microb Cell Fact. 2018 Nov 19;17:180. doi: 10.1186/s12934-018-1029-1 (PMC6240960; doi:10.1186/s12934-018-1029-1)
Supplement: Supplementary file 5 — Additional file 5. Table S1. Primers used for PCR amplification. [file 12934_2018_1029_MOESM5_ESM.docx]

**Additional file 5**

**Table S1.** **Primers used for PCR amplification**

| Primer name | Primer sequence (5′ to 3′) | Restriction site |
| --- | --- | --- |
| Δ-*gadA*-*U*-F | CGGGGTACCGACAACCTGGCAAACCAC | *Kpn*I |
| Δ-*gadA*-*U*-R | AAAACTGCAGGGATGATGCCTTTAGCCTG | *Pst*I |
| Δ-*gadA*-*D*-F | AAAACTGCAGTTAATAGGCAATGTAGTCGTCTA | *Pst*I |
| Δ-*gadA*-*D*-R | CTAGTCTAGACGAATAAAGATATTGATGATCTG | *Xba*I |
| Δ-*gadB*-*U*-F | CGGGGTACCACTCTTTCTATTTATTTTGTACCTCG | *Kpn*I |
| Δ-*gadB*-*U*-R | AAAACTGCAGCTACTTGGTTTCTTTTTCCAAC | *Pst*I |
| Δ-*gadB*-*D*-F | AAAACTGCAGGACCACCGTTCGAAGTTAA | *Pst*I |
| Δ-*gadB*-*D*-R | CTAGTCTAGACATAATCCAGTGGTTCCTGC | *Xba*I |
| Δ-*gadC*-*U*-F | CGGGGTACCGAATCCAGAACTAAGGAAAGG | *Kpn*I |
| Δ-*gadC*-*U*-R | ACGCGTCGACTTCAGACTTATTTTCATCCAC | *Sal*I |
| Δ-*gadC*-*D*-F | ACGCGTCGACTGAAAAATGATTAAGGAGGCAAGC | *Sal*I |
| Δ-*gadC*-*D*-R | CTAGTCTAGACCCATAGGCGTAAACAATCC | *Xba*I |
