# Supplementary material for: Exploring the contributions of two glutamate decarboxylase isozymes in Lactobacillus brevis to acid resistance and γ-aminobutyric acid production
Source: Microb Cell Fact. 2018 Nov 19;17:180. doi: 10.1186/s12934-018-1029-1 (PMC6240960; doi:10.1186/s12934-018-1029-1)
Supplement: Supplementary file 6 — Additional file 6. Table S2. Primers used for key GAD system genes amplification. [file 12934_2018_1029_MOESM6_ESM.docx]

**Additional file 6**

**Table S2. Primers used for key GAD system genes amplification**

| Primer name | Primer sequence (5′ to 3′) | Restriction site |
| --- | --- | --- |
| *gadCB*-F | AAAACTGCAGGCGTGGATGAAAATAAGTCTGAAC | *Pst*I |
| *gadCB*-R | CCCAAGCTTTTAACTTCGAACGGTGGTC | *Hin*dIII |
| *gadB*-F1 | AAAACTGCAGGCATGAATAAAAACGATCAGGAAAC | *Pst*I |
| *gadB*-R1 | CTAGTCTAGATTAACTTCGAACGGTGGTC | *Xba*I |
| *gadB*-F2 | ACGCGTCGACCATGAATAAAAACGATCAGGAAAC | *Sal*I |
| *gadB*-R2 | CCCAAGCTTTTAACTTCGAACGGTGGTC | *Hin*dIII |
| *gadC*-F | CTAGTCTAGAGAGTGGATGAAAATAAGTCTGAAC | *Xba*I |
| *gadC*-R | CCCAAGCTTCTACTTGGTTTCTTTTTCCAAC | *Hin*dIII |
