# Supplementary material for: Exploring the contributions of two glutamate decarboxylase isozymes in Lactobacillus brevis to acid resistance and γ-aminobutyric acid production
Source: Microb Cell Fact. 2018 Nov 19;17:180. doi: 10.1186/s12934-018-1029-1 (PMC6240960; doi:10.1186/s12934-018-1029-1)
Supplement: Supplementary file 7 — Additional file 7. Table S3. Primer used in the quantitative PCR. [file 12934_2018_1029_MOESM7_ESM.docx]

**Additional file 7**

**Table S3. Primer used in the quantitative PCR**

| Primers | Sequence (5’-3’) | Product size (bp) |
| --- | --- | --- |
| *gadR*-F | CGATTCCCATGCTTATTC | 114 |
| *gadR*-R | TTGCGGAAATGTAACTGC |  |
| *gadC*-F | TCTTAGTGGGATTTGTTCCG | 265 |
| *gadC*-R | agcttttcgacaaagaccac |  |
| *gadB*-F | aggcta atcaaaacctagcg | 264 |
| *gadB*-R | AACTATGTAGTAGCGCCAAG |  |
| *gadA*-F | ttgtagagcggtacaatcgg | 271 |
| *gadA*-R | ttgatggccatcgtaggtag |  |
| *16S rRNA*-F | tgagtgctaagtgttggagg | 267 |
| *16S rRNA*-R | acatctcacgacacgagctg |  |
